# Initial evaluation of the Celesteion large-bore PET/CT scanner in accordance with the NEMA NU2-2012 standard and the Japanese guideline for oncology FDG PET/CT data acquisition protocol version 2.0

**DOI:** 10.1186/s13550-017-0331-y

**Published:** 2017-10-11

**Authors:** Tomohiro Kaneta, Matsuyoshi Ogawa, Nobutoku Motomura, Hitoshi Iizuka, Tetsu Arisawa, Ayako Hino-Shishikura, Keisuke Yoshida, Tomio Inoue

**Affiliations:** 1 Department of Radiology, Yokohama City University, Yokohama, Japan; 2 Nuclear Medicine System Development Department, Toshiba Medical Systems Corporation, Tochigi, Japan

**Keywords:** PET, NEMA, TOF, Celesteion

## Abstract

**Background:**

The goal of this study was to evaluate the performance of the Celesteion positron emission tomography/computed tomography (PET/CT) scanner, which is characterized by a large-bore and time-of-flight (TOF) function, in accordance with the NEMA NU-2 2012 standard and version 2.0 of the Japanese guideline for oncology fluorodeoxyglucose PET/CT data acquisition protocol. Spatial resolution, sensitivity, count rate characteristic, scatter fraction, energy resolution, TOF timing resolution, and image quality were evaluated according to the NEMA NU-2 2012 standard. Phantom experiments were performed using ^18^F-solution and an IEC body phantom of the type described in the NEMA NU-2 2012 standard. The minimum scanning time required for the detection of a 10-mm hot sphere with a 4:1 target-to-background ratio, the phantom noise equivalent count (NEC_phantom_), % background variability (*N*_10mm_), % contrast (*Q*_H,10mm_), and recovery coefficient (RC) were calculated according to the Japanese guideline.

**Results:**

The measured spatial resolution ranged from 4.5- to 5-mm full width at half maximum (FWHM). The sensitivity and scatter fraction were 3.8 cps/kBq and 37.3%, respectively. The peak noise-equivalent count rate was 70 kcps in the presence of 29.6 kBq mL^−1^ in the phantom. The system energy resolution was 12.4% and the TOF timing resolution was 411 ps at FWHM. Minimum scanning times of 2, 7, 6, and 2 min per bed position, respectively, are recommended for visual score, noise-equivalent count (NEC)_phantom_, *N*_10mm_, and the *Q*_H,10mm_ to *N*_10mm_ ratio (QNR) by the Japanese guideline. The RC of a 10-mm-diameter sphere was 0.49, which exceeded the minimum recommended value.

**Conclusions:**

The Celesteion large-bore PET/CT system had low sensitivity and NEC, but good spatial and time resolution when compared to other PET/CT scanners. The QNR met the recommended values of the Japanese guideline even at 2 min. The Celesteion is therefore thought to provide acceptable image quality with 2 min/bed position acquisition, which is the most common scan protocol in Japan.

## Background

**Fig. 1:**
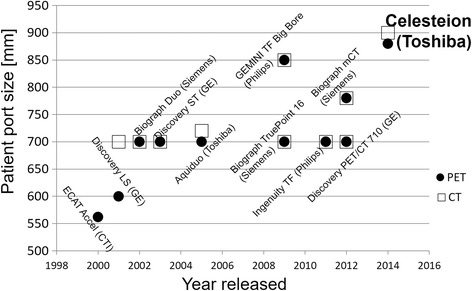
The size of the gantry opening for the CT and PET components. Adjacent black circles and squares correspond to the same scanner. Notice that the left-most black circle represents a dedicated PET scanner, not a PET/CT scanner. Data was obtained from Imaging Technology News (http://www.itnonline.com/)

The common size of the gantry opening (patient port size or bore size) of positron emission tomography/computed tomography (PET/CT) scanners for clinical use is approximately 700 mm (Fig. 1). This is adequate for most patients, although a larger bore may be necessary for overweight patients or for those requiring devices such as holding fixtures for radiotherapy or artificial ventilators.

Toshiba Medical Systems (Tochigi, Japan) has recently introduced the Celesteion, which is a large-bore PET/CT scanner (900 mm for the CT section and 880 mm for the PET section) that provides a wide-scan transaxial field of view (FOV) of up to 700 mm. The large bore improves patient comfort and allows the operator to observe and interact with the patient easily during the examination, which can help increase safety and decrease patient anxiety. However, the large-bore size may decrease the sensitivity of the PET scanner due to the decreased acceptance angle of the detected photons. Thus, the large bore negatively influences image quality.

In addition to the larger bore, the Celesteion has time-of-flight (TOF) capability. TOF provides information regarding the detected time difference between the annihilation pairs, which can be used to localize the annihilation point to a smaller region along the line of response. Thus, TOF-PET allows for much better localization of the original activity distribution of the radiopharmaceutical [1, 2]. This advantage of the technique is expected to compensate for the decrease in sensitivity due to the large bore in the Celesteion scanner*.*

The National Electrical Manufacturers Association (NEMA) has published a series of procedures used to evaluate the physical performance of PET systems [3, 4]. This NEMA standard is revised periodically, and the latest update of this publication resulted in the NEMA NU2-2012 standard. The purpose of this work was to evaluate the physical performance of the Celesteion PET/CT scanner according to the NEMA protocol NU-2 2012. We also calculated the timing and energy resolutions. Furthermore, we applied version 2.0 of the Japanese guideline for the oncology fluorodeoxyglucose (FDG) PET/CT data acquisition protocol [5]. This guideline evaluates the image quality using image-based noise equivalent count (NEC) and contrast of small hot lesions considering the most common injection dose in Japan (3.7 MBq/kg), which are thought to be interested mainly in oncology.

## Methods

### Scanner

The Celesteion PET/CT scanner combines a high-speed helical 16-slice CT scanner and a newly designed lutetium-yttrium oxyorthosilicate (LYSO) scintillator SUREFliGHT™ detector PET scanner [6]. The CT scanner has 16 rows of tube detectors with 994 detector cells per row, and each detector revolution can cover up to 32 mm.

The PET scanner contains LYSO scintillator crystals arranged in 48 rings. The crystals measure 4 mm transaxially by 4 mm axially and are arranged in detection units consisting of 48 × 16 crystals coupled to a photomultiplier. The 48-ring system can obtain 96 PET images (maximum ring difference, 47) spaced by 2.04 mm and covering an axial FOV of 19.6 cm. The PET scanner can acquire data in three-dimensional (3D) configurations. The energy window of the system is set to 425–650 keV, while the coincidence time window is set to 2.7 ns. The main technical features of the CT and PET components are summarized in Tables 1 and 2, respectively.

**Table 1:** Celesteion: major CT technical characteristics

Scan mode	Helical, conventional, dynamic, scanogram
Aperture (cm)	90
Maximum scan field of view (cm)	70
No. of slices per rotation	1–16
Nominal slice width (mm)	0.5, 1, 2, 3, 4, 6, 8
Tube voltage (kV)	80, 100, 120, 135
Tube current (mA)	10–600 in 10-mA increments
Detector material	Solid state
No. of elements along *Z*-axis	16
No. of detectors per row	994
Total effective length of detector array at isocenter (mm)	32
Variable scan speed (s)	0.5–1.5
Helical pitch	Detailed (11) Standard (15) Fast (23)
Max coverage (s/m)	10.9 (2 mm × 16 rows, HP:Fast, 0.5 s/rot.)
Maximum continuous scan time (s)	100
Helical interpolation algorithms available	MUSCOT and TCOT+ (helical)
Heat capacity (MHU)	7.5
Maximum power (kW)	72

**Table 2:** Celesteion: major PET technical characteristics

Detector ring diameter (cm)	90.7
Detector material	LYSO
No. of individual crystals	30,720
No. of crystals/ring	640
No. of detector rings	48
No. of image planes	96
Crystal size (mm^2^)	4 × 4
Face of crystal block (mm^2^)	196 × 65
Crystals per block	48 × 16
No. of PMTs	480
No. of detector modules	40
Patient port diameter (cm)	88
Axial field of view (cm)	19.6
Transaxial field of view (cm)	70
Axial sampling interval (mm)	2
Coincidence window width (ns)	1.6–3.2
Energy window (keV)	425–650
External pin source	^68^Ge (185 MBq)

*PMT* photomultiplier tube

### NEMA NU-2 2012 measurements

All measurements were performed at the site of the clinical installation of the system between September 2014 and December 2014, with the exception of those described in the “Image quality”, “Accuracy of attenuation”, and “Scatter corrections” sections of the NEMA protocol NU-2 2012, which were performed in October 2016.

### Spatial resolution

Spatial resolution was measured using a ^18^F solution point source in a glass capillary tube with an inner diameter of 0.8 mm. At the start of the measurement, the activity of the point source was 1.4 MBq. The axial extent of the activity in the tube was less than 1 mm. The source was placed at 3 transverse (*x*, *y*) positions: (0, 10), (0, 100), and (0, 200), and at 2 axial positions: *z* = 0 and *z* = 73.5 mm from the center of the axial FOV. List-mode data were acquired at each position and then reconstructed using Single-Slice Rebinning and Filtered Back Projection. The pixel size of axial image is 1 mm, and the slice thickness is 2 mm. No filters or correction methods, such as scatter correction, attenuation correction, point spread function, and TOF, were used. Profiles through the point source were obtained based on image data, and the full width at half maximum (FWHM) of each profile was determined using a quadratic fit.

### Sensitivity

Sensitivity was measured using a 700-mm-long plastic line source containing about 5.0 MBq of ^18^F solution at the start time of the scan. The measurements were obtained using a series of five concentric aluminum sleeves (defined in NEMA NU-2 2012). The line source was positioned parallel to the scanner axis and centered in the transverse and axial FOVs to within 2 mm. The measurements were also performed with a 10-cm radial offset. The data acquisition time was 300 s for each of the five measurements. Sensitivity was defined as the count rate per unit activity without attenuation and was obtained by extrapolating the measured values.

### Trues, scatter, randoms, and noise-equivalent count rate

A 700-mm-long, 3.2 mm-diameter plastic tube in a polyethylene scatter phantom (defined in NEMA NU-2 2012) was used to determine trues, scatter, randoms, and noise-equivalent count rate (NECR) curves. The tube was filled with 1049 MBq of ^18^F solution at the start of the scan. The phantom was positioned on the scanner’s table-top and centered in the FOV, which resulted in a line of activity 45 mm below the transverse center. Data were acquired for about 14 h in 120 frames (10 × 30-s frames, 20 × 60 s, 20 × 90 s, 20 × 120 s, 20 × 180 s, and 30 × 300 s; no inter-frame delay). Data from each interval were binned into a sinogram, and the count rates and scatter fraction were calculated. The NECR was calculated as described in NEMA NU-2 2012 using the formula for a system equipped with random estimation.

### Accuracies of count losses and random corrections

Accuracies of count losses and random corrections were measured using the same phantom used in the “trues, scatter, randoms, and noise-equivalent count rate” measurements, which contained 622 MBq of ^18^F solution at the scan start time. The data were corrected for random coincidences, normalization, dead time losses, scatter, and attenuation. The NEMA NU-2 2012 protocol clearly specifies the use of the standard whole-body algorithm. The 3 axial end slices (the first and last 3 slices) were excluded from the analysis. The maximum count rate error around the peak NECR and the maximum and minimum errors for all activity concentrations are reported.

### Image quality

A NEMA image quality (IQ) phantom containing six spheres with internal diameters of 10, 13, 17, 22, 28, and 37 mm was used for the evaluation of image quality. A cylindrical insert with a diameter of 5 cm containing a low-density material with an average density of 0.3 g/ml was positioned in the center of the phantom to simulate lung tissue and provide a non-uniform attenuation distribution (NEMA IEC Body Phantom; Data Spectrum; Hillsborough, NC, USA). This phantom was filled with ^18^F solution containing a background activity concentration of 5.3 kBq/ml. The four smallest spheres were filled with a target-to-background ratio (TBR) of 4:1. The two largest remaining spheres were filled with non-radioactive water. The phantom was positioned with all spheres aligned within the same transaxial image plane in the center of the FOV. To simulate a clinical situation with activity outside the FOV, the cylindrical phantom used for the count rate measurement was placed besides the IQ phantom as described in NEMA NU-2 2012. The line source for the scatter phantom was filled with 114 MBq of ^18^F solution at the start of the scan. Three sequential measurements of 240 s each were acquired for a single-bed position subsequent to a CT transmission scan for attenuation correction. All data were corrected for random coincidences, normalization, dead time losses, scatter, and attenuation. Data were reconstructed with TOF list-mode ordered subsets expectation maximization (TOF-LM-OSEM) using a 450 ps TOF temporal resolution kernel [7]. The TOF-LM-OSEM method is a TOF-OSEM algorithm using area-simulating volume, which calculates the geometric probabilities in the system matrix of 3D PET systems. For the reconstruction, a 208 × 208 matrix size (pixel size 2 mm) was used and a post-reconstruction Gaussian filtering with 6-mm FWHM was applied. The numbers of iterations and subsets were 3 and 10, respectively. The NEMA NU-2 2012 standard was followed to evaluate image quality. The average and range of percent contrast obtained for hot and cold spheres, the average and range of the standard deviation of the background counts, and the mean residual error for the scatter and attenuation corrections were evaluated.

### TOF timing resolution and energy resolution

To measure the TOF timing resolution and energy resolution, which are parameters not included in the scope of NEMA, data were collected using the ^68^Ge rod source (185 MBq) positioned within the 2-mm radius of the scanner’s isocenter. The energy and TOF difference for each detected coincidence were obtained from the data and accumulated into histograms. The energy resolution and timing resolution were then defined as the FWHM of the energy histogram and the time difference histogram, respectively. Gaussian fitting was used to calculate the FWHMs.

### Japanese guideline for oncology FDG-PET/CT data acquisition protocol

The Japanese guideline for oncology FDG-PET/CT data acquisition protocol [5] requires two phantom experiments (#1 and #2). Experiment #1 is carried out to determine the minimum scan duration for the detection of a 10-mm-diameter hot sphere with a TBR of 4:1. The images were displayed using an inverse gray scale with an upper standardized uptake value (SUV) level of 4 and a lower SUV level of 0. The image was given a score of 2 when the hot sphere was “identifiable”, a score of 1 when it was “visualized, but similar hot spots were observed elsewhere”, and a score of 0 if it was “not visualized”. The scores were averaged across the three image sets for each scanning duration and across physicians. Experiment #1 also requires the calculation of the phantom noise equivalent count (NEC_phantom_), % background variability (*N*_10mm_), and % contrast (*Q*_H,10mm_). In experiment #2, six spheres (inner diameters of 37, 28, 22, 17, 13, and 10 mm) in the phantom were filled with an ^18^F solution with a TBR of 4:1 under noise-free conditions in order to estimate image resolution based on the recovery coefficient (RC). The ^18^F-solution and IQ phantom used to determine “image quality” as described in the NEMA NU-2 2012 standard were used for these experiments. The Japanese guideline recommends using scanning durations that provide images with average scores of 1.5 or higher. The values of the above described parameters were as follows: NEC_phantom_ > 10.8 Mcounts, *N*_10mm_ < 5.6%, QNR > 2.8, and RC of a 10-mm-diameter sphere > 0.38. These values were used as reference values.

### Data acquisition and reconstruction conditions

The experiments were performed as described in the Japanese guideline [3]. The background activity was 2.65 kBq/mL, which is closer to Japanese FDG PET study (a common injected dose of 3.7 MBq/kg of weight) rather than 5.30 kBq/mL recommended in the NEMA NU-2 2012 standard. The scan time for PET was varied from 1 to 10 min in 1-min steps. CT scanning was performed using 120 kV of tube voltage. We used a 200 mA tube current. The FOV was set to 550 mm.

PET images were reconstructed using the TOF-LM-OSEM method using a 450 ps TOF temporal resolution kernel. The matrix size was 144, the pixel size was 4 mm, and the slice thickness was 4 mm. The scatter and random corrections were performed using simulation-based scatter correction method and delayed coincidence and spatial filtering method, respectively. The scatter and spatial filtered random estimates are included in the OSEM system matrix.

PET data obtained using a 2-min scan were used to find the optimal reconstruction parameters. The number of iterations was varied from 1 to 5 in the TOF-LM-OSEM reconstruction. The number of subsets was set to 10, as described in a previous study [7]. The Gaussian filter parameters (FWHM) were 3, 6, and 9 mm. The QNR was calculated for each of these parameters. The optimal reconstruction parameters were defined as those that provided the maximum QNR using the 2-min acquisition images. These parameters were then used in the reconstruction of PET images acquired using other scan times.

### Indicators of phantom image quality

The images acquired in the phantom experiments were evaluated using the following indicators:

1. Phantom noise equivalent count (NEC_phantom_), which was calculated as follows:

$$ {\displaystyle \begin{array}{c}\mathrm{NEC}\left[\mathrm{Mcounts}\right]=\frac{T^2}{T+S+\left(1+k\right) fR}\\ {}={\left(1-\mathrm{SF}\right)}^2\frac{{\left(P-D\right)}^2}{\left(P-D\right)+\left(1+k\right) fD}\end{array}} $$

1 $$ f=\frac{S_{\mathrm{a}}}{\uppi {r}^2} $$

where *T*, *S*, and *R* represent true, scatter, and random coincidences acquired within the scanning period and *P* and *D* represent prompt and delayed coincidences. SF, *k*, and *f* represent scatter fraction, random scaling factor, and the percentage of the cross-sectional area of the phantom occupying the cross-sectional area of the FOV for imaging, respectively. Here, *S*_a_ is a cross-sectional area of the phantom (cm^2^) and *r* is 1/2 of the distance between the cross-section and the detector (cm) (each body phantom has a different cross-sectional area). SF will be determined from the results of the NEMA NU-2 2012 measurements.

2. Percent contrast (*Q*_H,10mm_)

The % contrast for the 10-mm hot sphere (*Q*_H,10mm_) was calculated as follows:

2 $$ {Q}_{\mathrm{H},10\mathrm{mm}}=\frac{C_{\mathrm{H},10\mathrm{mm}}/{C}_{B,10 mm}-1}{a_H/{a}_B-1}\times 100\% $$

where *C*_H,10mm_ indicates the average measured counts in the ROI for the 10-mm sphere. *C*_B,10mm_ indicates the average measured counts in 60 10-mm-diameter ROI set in background regions (12 ROIs in each five slice). *a*_H_ indicates the measured activity concentration in the hot spheres, and *a*_B_ indicates the one in the background.

3. Percent background variability (*N*_10mm_)

The % background variability (*N*_10mm_) for the 10-mm sphere was calculated as follows:

3 $$ {N}_{10\mathrm{mm}}=\frac{SD_{10\mathrm{mm}}}{C_{B,10\mathrm{mm}}}\times 100\% $$

where SD_10mm_ is the standard deviation of the background, which is calculated as follows:

4 $$ {\mathrm{SD}}_{10\mathrm{mm}}=\sqrt{\frac{\sum_{K=1}^K{\left({C}_{b,10\mathrm{mm},k}-{C}_{B,10\mathrm{mm}}\right)}^2}{K-1}},K=60 $$

where *K* indicates the number of 10-mm-diameter ROI set in the background regions.

4. RC

The recovery coefficient for a *j* mm-diameter hot sphere (RC_*j*_) is the quotient of the maximum pixel value (C_*j*_) within the ROI over the sphere on the reconstructed image and the maximum pixel value of a 37-mm-diameter sphere (C_37_), i.e., $$ {\mathrm{RC}}_j=\raisebox{1ex}{$\ {C}_j$}\!\left/ \!\raisebox{-1ex}{${C}_{37}$}\right.. $$ These values were calculated for images corresponding to the 30-min acquisition experiment in order to reduce statistical noise.

### Patient study

The institutional review board (IRB) of our university approved the use of patient data for this study. Informed consent was waived by the IRB due to the retrospective nature of the study. The PET data obtained using list-mode were reconstructed for scans with 90, 120, and 150 s/bed position. These images were evaluated visually and quantitatively. For the quantitative analysis, the volumes of interest were placed in the liver and mediastinum (3 cm in diameter) and in the brain (2 cm in diameter), to measure the parenchyma of the organs. The maximum SUV and mean SUV were calculated. Using images with 120 s/bed position, NECpatient (noise equivalent count per patient height) and NECdensity (noise equivalent count per volume) were calculated as potential physical indicators of image quality. The liver signal-to-noise ratio (SNR) was also computed as mean/SD within the liver ROI, which was located away from the porta hepatis and major vessels in the three coronary sections [5].

## Results

### Spatial resolution, sensitivity, and count rate

**Fig. 2:**
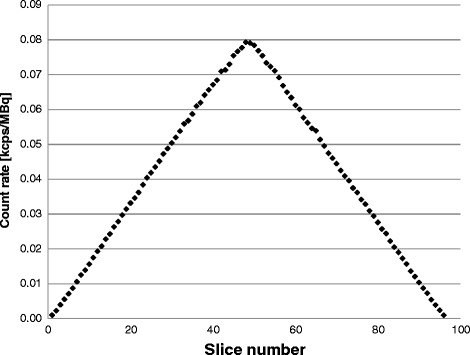
The axial sensitivity profile with the line source placed at the center of the FOV

The calculated spatial resolution is shown in Table 3. Each point source position was within a 2-mm radius of the desired position, as measured using its 3D centroid in the image. The slice sensitivity profiles were triangular (Fig. 2), as is expected in a scanner with a 3D geometry and no axial-angle restriction. The measured sensitivity was 3.8 cps/kBq for both, the 0- and 10-cm off-center positions.

**Table 3:** Spatial resolution measured for the PET component of the Celesteion system

Spatial resolution	Distance (mm)	Measured FWHM (mm)	Product specification^a^ FWHM (mm)
Transverse radial	10	4.5	< 5.0
Transverse tangential	10	4.7	< 5.1
Axial	10	4.4	< 5.1
Transverse radial	100	4.6	< 5.4
Transverse tangential	100	4.8	< 5.2
Axial	100	4.6	< 5.1
Transverse radial	200	5.8	< 6.1
Transverse tangential	200	5.3	< 5.8
Axial	200	4.7	< 5.2

^a^Provided by Toshiba Medical Systems

**Fig. 3:**
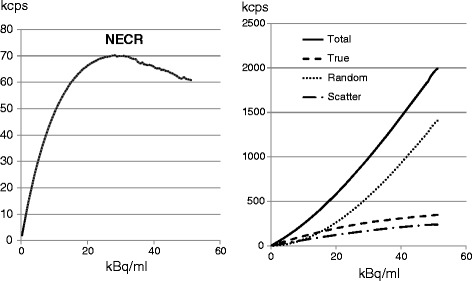
The activity-rate curves. The curves of total, true, random, and scatter events are shown on the right and that of NECR is shown on the left. The peak NECR was 70 kcps when the phantom contained 29.6 kBq mL^−1^

The peak NECR was 70 kcps when the phantom contained 29.6 kBq/ml. The true event rate was 220 kcps. The scatter fraction was 37.3% at the peak NECR and ranged from 33% at low count rates up to a maximum of 39% at an activity concentration of 45 kBq/ml (Table 4 and Fig. 3).

**Table 4:** Sensitivity and count rates of the Celesteion system

	Measured	Product specifications^a^
Sensitivity	3.8 cps/kBq	≥ 3.6 cps/kBq
Count rate peak NECR	70 kcps	61 ± 10 kcps
Count rate peak true	220 kcps	≥ 180 kcps
System energy resolution	12.4%	≤ 13.7%
TOF timing resolution	411 ps	≤ 450 ps
Scatter fraction (weighted)	37.3%	≤ 42.7%

^a^Provided by Toshiba Medical Systems

### Count rate accuracy

**Fig. 4:**
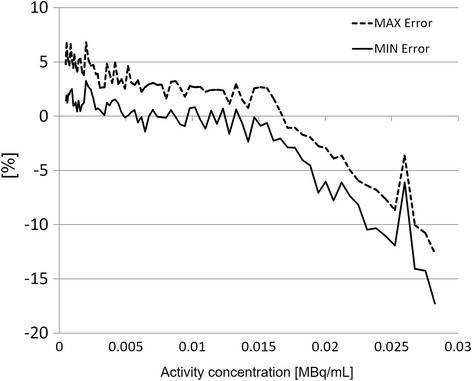
Maximum and minimum relative count rate errors for the different activity distributions. The dashed line shows the maximum value; the solid line shows the minimum value. The first 3 and the last 3 slices of the acquisitions were excluded from this evaluation

The relative count rate error at the activity concentration of the NECR peak (29.6 kBq/ml) was 17.3%. The maximum and minimum errors for all activity concentrations are depicted in Fig. 4.

### Reconstruction conditions

**Fig. 5:**
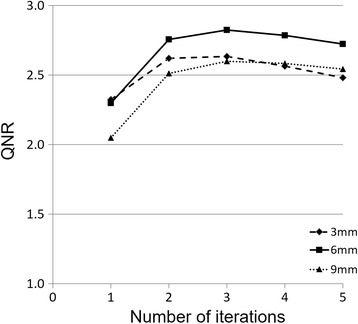
Changes in the *Q*_H,10mm_ to *N*_10mm_ ratio (QNR) as a function of the number of iterations for different Gaussian filters. Of the first to fifth iterations, and 3, 6, and 9 mm FWHM values for the Gaussian filters were used. The maximum QNR (2.82) was found at 3 iterations and for the 6-mm Gaussian filter FWHM

As shown in Fig. 5, the optimal number of iterations was 3 and the optimum FWHM for the Gaussian filter was 6 mm, which resulted in a maximum QNR value of 2.82. We thus used these conditions for image reconstruction.

### Image quality phantom (hot spheres at the center of the axial FOV)

**Fig. 6:**
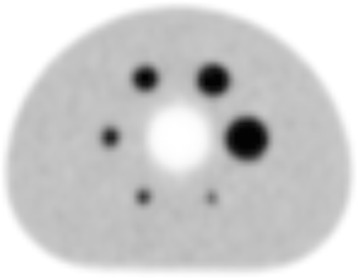
Central slice of the image quality phantom. The TBR was 4:1

The contrast, background variability, and average residuals in the lung that were computed from these images are shown in Table 5. The transaxial section through the center of the spheres within the image quality phantom is shown in Fig. 6 for the scan using a TBR of 4:1.

**Table 5:** Image quality results for a TBR of 4:1

Sphere diameter (mm)	Contrast recovery	Background variability
10	27.9	6.2
13	48.6	5.3
17	52.0	4.6
22	60.5	4.1
28	72.9	3.9
37	77.1	3.8

The average residual value in the lung was 5.0%

### TOF timing resolution and energy resolution

The timing and energy resolutions measured using the ^68^Ge rod source were 411 ps FWHM and 63.4 keV FWHM (12.4%), respectively.

### Minimum scan duration for detection of a 10-mm-diameter hot sphere

Two board-certified physicians read the images. The average score indicated that 2 min/bed position would be required to meet the score recommended by version 2.0 of the Japanese guideline (> 1.5).

### NEC_phantom_, *Q*_H,10mm_, *N*_10mm_, QNR, and RC

**Fig. 7:**
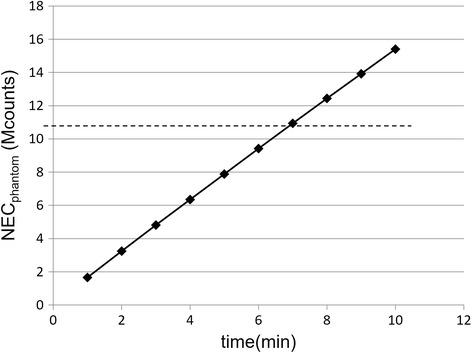
Noise equivalent counts (NEC_phantom_) measured for 1–10 min of scanning time (1-min intervals). The dotted line indicates the value recommended in the Japanese guideline (10.8 Mcounts)

The NEC_phantom_ results (Fig. 7) indicated that 7 min/bed position would be required to meet the value recommended by version 2.0 of the Japanese guideline (> 10.8 Mcounts).

**Fig. 8:**
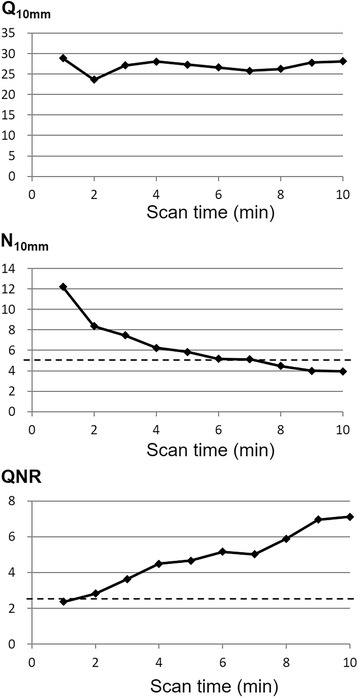
The relationships of the % contrast (*Q*_H,10mm_), the % background variability (*N*_10mm_), and the QNR with different scan times. The dotted lines indicate the recommended values (*N*_10mm_ < 5.6%, QNR > 2.8)

**Fig. 9:**
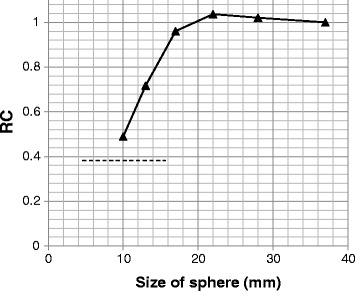
The recovery coefficients (RC) measured for different sphere sizes. Images with 10-min acquisition were used. The dotted line indicates the value recommended in the Japanese guideline (> 0.38)

The *N*_10mm_ value was below the maximum recommended value (< 5.6%) at 6 min, and the QNR exceeded the minimum recommended value (> 2.8) at 2 min. The RC of a 10-mm-diameter sphere was 0.49, which indicates that a much sharper image was obtained than required by the recommended value (> 0.38). These measurements are shown in Figs. 8 and 9.

### A patient study

**Fig. 10:**
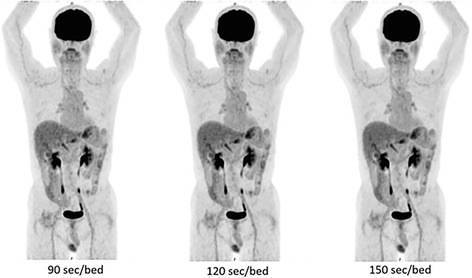
Sample clinical images with different acquisition times. This patient is a 71-year-old man with a height of 159 cm and a weight of 53.2 kg. Left: 90 s/bed position, middle: 120 s/bed position, right: 150 s/bed position. The PET scan was performed 63 min after the injection of 192.8 MBq (3.62 MBq/kg)

Figure 10 shows the images obtained from a male patient after treatment for intrahepatic bile duct cancer (age, 71 years; height, 159 cm; weight, 53.2 kg).

The PET scan was performed 63 min after the injection of 192.8 MBq (3.62 MBq/kg) of FDG. The blood sugar level just before the injection of FDG was 78 mg/dl. No visual difference was observed by two experienced imaging experts among the images reconstructed for 90, 120, and 150 s/bed position. Nonetheless, increased noise was observed in images acquired using 90 s/bed position (left panel in Fig. 9). The SUVmax and SUVmean in the liver, mediastinum, and brain are provided in Table 6. The differences in SUVmax and SUVmean among the three images were equal to or less than 0.12 in the liver, 0.08 in the mediastinum, and 0.56 in the brain.

**Table 6:** SUVmax and SUVmean values of reference regions in the patient scans

	SUVmax [relative (%) compared to 180 s]			SUVmean (SD^a^) [relative (%) to 180 s]		
Organ	90 s	120 s	180 s	90 s	120 s	180 s
Liver	2.78 [100.0]	2.90 [104.3]	2.78	1.97 (0.22) [90.8]	2.09 (0.22) [96.3]	2.17 (0.25)
Mediastinum	1.74 [100.0]	1.78 [102.3]	1.74	1.28 (0.19) [100.0]	1.33 (0.18) [103.9]	1.28 (0.13)
Brain	9.00 [95.4]	9.41 [99.8]	9.43	7.34 (1.33) [87.3]	7.97 (1.20) [94.8]	8.41 (0.98)

^a^SD of pixel values within the ROI

The volumes of interest were placed in the liver and mediastinum (3 cm in diameter) and in the brain (2 cm in diameter) to measure the parenchyma of the organs. The number in parenthesis indicates the standard deviation of SUVmean.

**Fig. 11:**
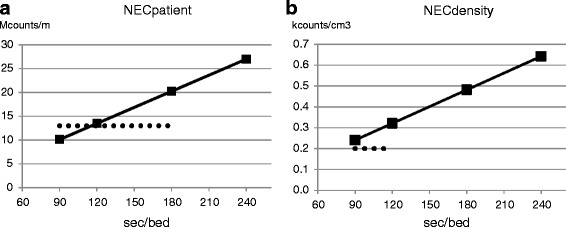
NECpatient (**a**) and NECdensity (**b**) values of the images reconstructed for 90, 120, 180, and 240 s/bed position. The dotted lines indicate the recommended values (NECpatient > 13 Mcounts/m, NECdensity > 0.2 kcounts/cm^3^)

NECpatient and NECdensity values are shown in Fig. 11. The NECpatient values indicated that 2 min/bed position would be required to meet the value recommended by version 2.0 of the Japanese guideline (> 13 Mcounts/m). The values of NECdensity indicated that 90 s/bed position would be required to meet the guideline (> 0.2 kcounts/cm^3^). The liver SNR was calculated to be 25.7 for 90 s/bed position, 32.4 for 120 s/bed position, and 72.3 for 150 s/bed position. All these values exceed the value required to meet the guideline (> 10).

## Discussion

In this study, we followed the NEMA NU-2 2012 standard and the Japanese guideline and evaluated the performance of the Celesteion PET/CT scanner, which is characterized by a large bore and TOF function. Our results are useful in comparing this system to other systems in providing us with an idea of the relative strengths and weaknesses of this device. To our knowledge, there is only one study reporting the results of a performance evaluation based on the NEMA NU2-2012 standard using the Biograph mCT Flow PET/CT system (Siemens Medical Solutions USA, Inc.) [8]. Our results demonstrated that, compared to the latest scanner described here, the spatial resolution (FWHM) of the mCT at the center seems to be equivalent (axial: 4.4 mm for Celesteion and 4.3 mm for mCT). FWHM at 10 cm was slightly better in the new device than in the mCT (axial: 4.6 mm for Celesteion and 5.9 mm for mCT). Finally, FWHM at 20 cm was much better in the new device than in the mCT (axial: 4.7 mm for Celesteion and 7.8 mm for mCT) [8]. The better off-center spatial resolution may be due to the decrease in the depth-of-interaction effect due to the large bore and the thin scintillators. The sensitivity and the maximum NECR were lower in the Celesteion than in the latest scanners (mCT: 185 kcps at 29.0 kBq/ml) [8]. These findings can also be explained by the decreased acceptance angle due to the large bore and by the increase in the number of penetrating photons due to the thin scintillators.

Our measured NEC_phantom_ values for the Celesteion were lower than those for other PET scanners, as measured by the working group of the Japanese Society of Nuclear Medicine Technology (http://www.jsnmt.umin.ne.jp/contents/guideline/GL_petscan.pdf [in Japanese]) [5]. This metric does not take any reconstruction characteristics (such as TOF information) into account. At least 7 min/bed position was necessary to achieve the minimum recommended value of the guideline (NEC_phantom_ > 10.8 Mcounts). To compensate this disadvantage, the Celesteion now equips TOF function with high temporal resolution. In addition, the relative count rate error at the activity concentration of the NECR peak was high (17.3%). The Celesteion might be designed to give priority to the temporal resolution to enhance the effect of TOF, but not to sensitivity at high count rates. This may lead to considerable errors for high count rates when using very short half-life radioisotopes. However, it seems not to be a problem for clinical FDG PET.

The NEC, which is defined in the NEMA NU-2 2012 standard, is an automatic and objective parameter that is determined from the reconstructed image or the raw positron emission data of phantoms. However, Conti [9] has proposed the use of an effective NEC that accounts for the TOF (NEC_TOF_). The gain in NEC due to TOF is proportional to the size of the object being imaged and inversely proportional to the temporal resolution. Thus, for an object that is 30 cm in diameter, the gain is about 4.9 with a temporal resolution of 411 ps, as follows: gain = 30 cm/(411 ps × (*c*/2)) = 4.9, where *c* is the velocity of light. Considering the TOF gain, the NEC_phantom_ parameter is changed from 7 min/bed position to 1.5 min/bed position (7 min/bed position is divided by the gain of 4.9). In this case, it is possible to achieve the NEC_phantom_ parameter recommended by the Japanese guideline with less than 2 min/bed position.

NEC is a metric that does not consider the impact of the reconstruction method used. Thus, the NEC is not a good evaluation index for image quality, although it is a good index to evaluate raw data quality [10]. Other indicators, such as *N*_10mm_ and *Q*_H,10mm_, might be more suitable than NEC for evaluating the image quality of TOF PET. The *N*_10mm_ and *Q*_H,10mm_ are the parameters which evaluate the image noise and image signal, respectively. Due to these differences, these parameters may meet the Japanese guideline at different time/bed. The QNR is a signal-to-noise ratio which represents image quality. This image quality is thought to be the most important for clinical image reading especially in oncology.

Our *N*_10mm_ results suggest that at least 6 min/bed position are required for the Celesteion device to meet the recommendation of the Japanese guideline (*N*_10mm_ < 5.6%). This scanning time seems too long for clinical practice. Additional noise reduction techniques might be necessary to solve this problem.

Our QNR results indicate that 2 min/bed position are sufficient to meet the minimum recommendation of the Japanese guideline (QNR > 2.8). As shown in Fig. 9, the image obtained using 90 s/bed position looks a little noisy while those obtained using 120 or 150 s/bed position do not. The image obtained from 2 min/bed is thought to be of sufficient visual quality. In addition, the differences in SUVmax and SUVmean among the three images were small. The standard deviation of the SUVmean of the mediastinum and brain showed a decreasing tendency for longer acquisition time, but the differences were also small (Table 6). Considering the average height of the Japanese adult (approximately 170 cm for men, 160 cm for women), about nine bed positions are required for whole body scanning. Thus, an examination could be completed within 30 min including the entrance, positioning, and leave, which would be preferable for routine clinical use.

## Conclusions

The Celesteion large-bore PET/CT system had low sensitivity and NEC when compared to the latest scanner. However, thanks to the incorporation of TOF information, the QNR met the recommended values of the Japanese guideline even for scan times of 2 min. Considering the common scan protocol in Japan, which requires 2 min/bed position or less, the Celesteion is thought to provide acceptable image quality in daily practice. The use of advanced reconstruction methods or imaging filters may help us to achieve better image quality and shorter scan times.

## Abbreviations

3D: Three-dimensional
FDG: Fluorodeoxyglucose
FOV: Field of view
IQ: Image quality
IRB: Institutional review board
LM: List-mode
LYSO: Lutetium-yttrium oxyorthosilicate
*N*_10mm_: % background variability
NEC: Noise equivalent count
NEC_phantom_: Phantom noise equivalent count
NECR: Noise-equivalent count rate
NEMA: National Electrical Manufactures Association
OSEM: Ordered subsets expectation maximization
PET/CT: Positron emission tomography/computed tomography
*Q*_H,10mm_: % contrast
QNR: *Q*_H,10mm_ to *N*_10mm_ ratio
RC: Recovery coefficient
SNR: Signal-to-noise ratio
SUV: Standardized uptake value
TBR: Target-to-background ratio
TOF: Time-of-flight
